# Are simple verbal instructions sufficient to ensure that bladder volume does not deteriorate prostate position reproducibility during spot scanning proton therapy?

**DOI:** 10.1259/bjro.20210064

**Published:** 2021-11-11

**Authors:** Kentaro Nishioka, Kento Gotoh, Takayuki Hashimoto, Takashige Abe, Takahiro Osawa, Ryuji Matsumoto, Isao Yokota, Norio Katoh, Rumiko Kinoshita, Koichi Yasuda, Toshiaki Yakabe, Takaaki Yoshimura, Seishin Takao, Nobuo Shinohara, Hidefumi Aoyama, Shinichi Shimizu, Hiroki Shirato

**Affiliations:** 1 Department of Radiation Medical Science and Engineering, Faculty of Medicine, Hokkaido University, Sapporo, Hokkaido, Japan; 2 Department of Radiation Medical Science and Engineering, Radiation Medical physics, Hokkaido University, Sapporo, Hokkaido, Japan; 3 Department of Renal and Genitourinary Surgery, Hokkaido University Graduate School of Medicine / School of Medicine, Sapporo, Hokkaido, Japan; 4 Department of Biostatistics, Hokkaido University Graduate School of Medicine, Sapporo, Hokkaido, Japan; 5 Department of Radiation Oncology, Faculty of Medicine, Hokkaido University, Sapporo, Hokkaido, Japan; 6 Department of Radiation Oncology, Hokkaido University Hospital, Sapporo, Hokkaido, Japan; 7 Department of Health Sciences and Technology, Faculty of Health Sciences, Hokkaido University, Sapporo, Hokkaido, Japan; 8 Global Center for Biomedical Science and Engineering, Faculty of Medicine, Hokkaido University, Sapporo, Hokkaido, Japan

## Abstract

**Objectives:**

The purpose of this study is to investigate whether verbal instructions are sufficient for bladder volume (BV) control not to deteriorate prostate position reproducibility in image-guided spot scanning proton therapy (SSPT) for localized prostate cancer.

**Methods:**

A total of 268 treatment sessions in 12 consecutive prostate cancer patients who were treated with image-guided SSPT with fiducial markers were retrospectively analyzed. In addition to strict rectal volume control procedures, simple verbal instructions to void urine one hour before the treatment were used here. The BV was measured by a Bladder Scan just before the treatment, and the prostate motion was measured by intraprostatic fiducial markers and two sets of X-ray fluoroscopy images. The correlation between the BV change and prostate motion was assessed by linear mixed-effects models and systematic and random errors according to the reproducibility of the BV.

**Results:**

The mean absolute BV change during treatment was from −98.7 to 86.3 ml (median 7.1 ml). The mean absolute prostate motion of the patients in the left-right direction was −1.46 to 1.85 mm; in the cranial-caudal direction it was −6.10 to 3.65 mm, and in the anteroposterior direction −1.90 to 5.23 mm. There was no significant relationship between the BV change and prostate motion during SSPT. The early and late genitourinary and gastrointestinal toxicity was minimal with a minimum follow up of 4.57 years.

**Conclusions:**

Simple verbal instructions about urination was suggested to be sufficient to control the BV not to impact on the prostate motion and clinical outcomes in image-guided SSPT. Careful attention to BV change is still needed when the seminal vesicle is to be treated.

**Advances in knowledge:**

Our data demonstrated that there was no apparent relationship between BV changes and prostate position reproducibility and simple verbal instruction about urination could be sufficient for image-guided SSPT.

## Introduction

External-beam radiotherapy is an established treatment for localized prostate cancer.^1^ Proton beam therapy (PBT) is an evolving external-beam radiotherapy with superior dose conformation at the Bragg peak followed by the steep gradient at the end of its range.^2^ This allows reductions in the total dose reaching non-target structures without compromising tumor dose coverage.^3^ Spot-scanning proton therapy (SSPT) has become a cutting-edge treatment for prostate cancer improving the dose distribution of PBT further with or without intensity modulation.^4^

Since the radiotherapy is carried out based on treatment planning computed tomography (TPCT) acquired before the treatment, there is a need to ensure that differences in the prostate position between the treatment plan and the actual position are minimal. Recent advances in image guidance techniques have improved treatment accuracy and clinical outcomes.^5–8^ However, in image-guided SSPT, the water equivalent length of the proton beam pathway from the skin surface to the target may change with volume changes of organs near the prostate and consequently larger margins may be required for robustness.^9–13^ Improvement of prostate position reproducibility in the SSPT is more important than in intensity modulated X-ray therapy (IMXT).

Rectal volume changes affect the position of the prostate.^14–16^ However, the importance of considering bladder volume (BV) changes on prostate position is not commonly agreed on. Previous studies that evaluated the effect of bladder filling on dose-volume statistics in prostate X-ray therapy reported that an increase in the doses to the bladder and bowel loops occurred as a result of treatment plans with small BV.^17,18^ In the real world, considerations to keep a full bladder and the BV constant during fractionated radiotherapy are widely recommended and verbal or written instructions for the patients about the timing of urination and water intake are commonly used without further intervention.^19–23^ However, the compliance to the instructions is not certain, the amount of urine accumulated within a certain period of time can vary among individuals, and daily BV changes can be large even in the same patient.^24^ From the aspect of value-based health care, strict instruction to the patient about BV control may or may not be recommendable depending on the clinical outcomes after the radiotherapy. In clinical practice, it is important to know whether simple verbal instructions to the patient are sufficient to keep BV changes below the threshold, if one has been set, impacting on the prostate motion also with the precise image-guided radiotherapy. The clinical outcomes and the relationship between BV changes and prostate movements have been reported in the setting of the X-ray therapy; however, these data and the effectiveness of instructions to control BV in the proton setting have not been reported previously.

This study investigated whether simple verbal instructions before radiotherapy are sufficient to maintain a modestly full bladder and keep the variation of BV within limits not to deteriorate prostate position reproducibility and clinical outcomes in image-guided SSPT. Then, correlations between BV and prostate motion and the threshold of BV variations which could improve prostate position reproducibility were investigated.

## Methods

### Patients

A total of 268 treatment sessions in 12 consecutive prostate cancer patients treated with image-guided SSPT in our hospital between April and August 2016 were retrospectively analyzed. No patients had received surgical intervention or radiotherapy before the SSPT. Genitourinary (GU) and gastrointestinal (GI) toxicities were evaluated based on the National Cancer Institute Common Terminology Criteria for Adverse Events (NCI-CTCAE) v.4.0. Acute and late toxicities were defined as side effects occurring within 90 days and after 90 days from the start of the proton therapy. This study was approved by the Institutional Review Board of our facility for clinical research (016–0210).

### Treatment preparation protocol and equipment

Three or four spherical 1.5-mm-diameter fiducial markers were implanted percutaneously in the prostatic gland about one week prior to the TPCT. The TPCT images were acquired with a slice thickness of 1.25 mm from the supine position for all patients using Optima CT580W (GE Healthcare, Waukesha, WI). A vacuum cushion was used to set the patient body and maintain the location of the legs. The patients were verbally instructed to void urine and stool as far as possible and to refrain from urination for one hour before the TPCT. There were neither restrictions nor recommendations for diet or drinking of water before the TPCT. We prescribed laxatives and/or probiotics to patients with constipation. When residual stool or gas was observed in the rectum on the CT images, we evacuated this by catheter or asked patients to void and then acquired images again.

Similarly, instructions for urination and defecation one hour before the treatment were given verbally before each treatment. If residual gas was observed in the patient rectum on the X-ray fluoroscopy on the patient couch at the PBT, the treatment was carried out after evacuation. If the prostate displacement relative to the bone structure determined by two orthogonal X-ray images was more than 5 mm in any direction and 4 mm in the posterior direction, a cone-beam CT (CBCT) was acquired to clarify the cause of the prostate motion. To evaluate the volume change of the rectum, we compared the average rectal cross-sectional area (CSA) on TPCT and CBCT. The average CSA was defined as the average of the rectal area contoured at the level of the prostate.

An image-guided SSPT system (PROBEAT-RT, Hitachi, Ltd., Tokyo) was used for the treatment. Two sets for X-ray fluoroscopy and a six-degree robotic patient couch are used for the precise set-up using bone structures, fiducial markers, and CBCT as described above.

### Treatment

The risk of recurrence was classified using the National Comprehensive Cancer Network (NCCN) risk classification.^25^ In cases where the risk of recurrence was lower than the favorable intermediate risk, only the prostate was defined as the clinical target volume (CTV), whereas in cases where the risk of recurrence was higher than the unfavorable intermediate risk, the seminal vesicle (SV) was also included as CTV in addition to the prostate.

**Figure 1.**
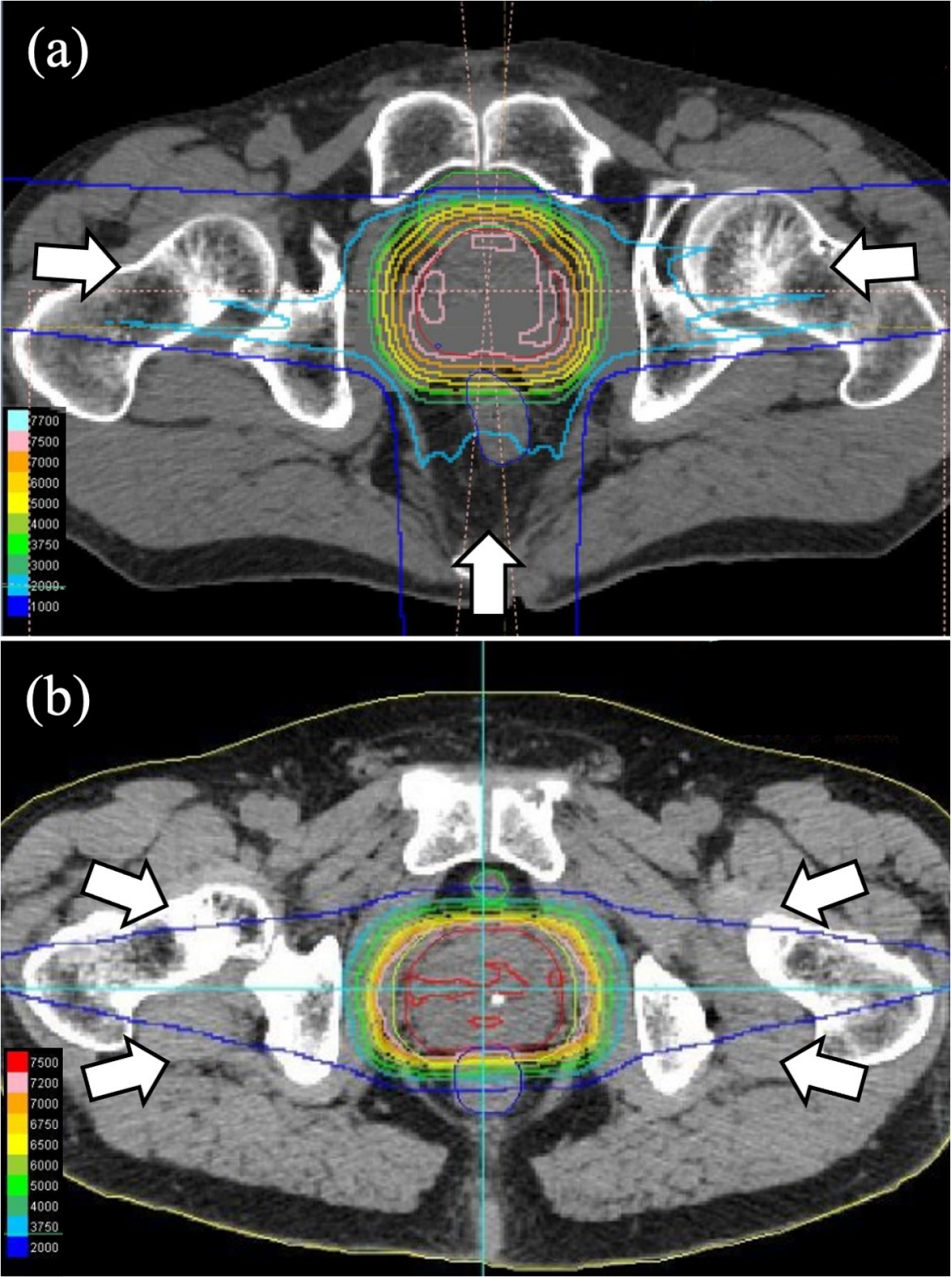
The representative dose distribution for the 3 (**a**) and 4 (**b**) field beam arrangements. The white arrows indicate the direction of proton beams.

The dose constraints for each target and organ at risk (OAR) are shown in Table 1. Patients whose OAR dose constraints were difficult to achieve with a single field uniform dose (SFUD) were treated with intensity-modulated proton therapy (IMPT). For SFUD, a 3 mm margin that encompassed the internal and setup margins was added laterally to the beam direction. Distal and proximal margins, which were calculated as 3.5% of the distal or proximal range plus 1 mm, respectively, were assigned to account for range uncertainties. For the IMPT planning, robust optimization assuming a setup error of 3 mm and a range uncertainty of 3.5% was used. In addition, the CTV was geometrically expanded by 4 mm to generate the planning target volume (PTV) for the purpose of the plan evaluation. The relative biological effectiveness (RBE) value was determined to be 1.1 by a previous study and used in the treatment planning.^26^ Seventy Gy (RBE) was prescribed to 99% of the CTV as well as 95% of the PTV in 30 fractions over 7.5 weeks for all patients. Fiducial markers placed in the prostate may cause dose distortions in proton therapy with a limited number of fields^27^ and therefore the treatment was performed with three fields from the left and right and the back, or four fields form either side of the slightly anterior and posterior oblique fields. The representative dose distributions for the 3 and 4 field beam arrangements are shown in Figure 1. The beam directions are determined, as far as possible, not to pass through the intestine and bladder, which may change volume daily.

**Table 1.** Dose constraints for target and organs at risk

Volume	Parameter	Criteria	Acceptable criteria
**PTV**	**D95 (Gy(RBE**))	70 Gy(RBE)	-
**CTV**	**D99 (Gy(RBE**))	>70 Gy(RBE)	-
	**Dmax (Gy(RBE**))	<77 Gy(RBE)	<80.5 Gy(RBE)
**Rectum**	**V60 Gy(RBE**)	<20%	<30%
	**V37.5 Gy(RBE**)	<50%	-
**Bladder**	**V37.5 Gy(RBE**)	<30%	<50%

CTV: clinical target volume, D95(99): the dose covering 95(99) % of the target, Dmax: max dose, V60(37.5) Gy(RBE): the volume receiving more than 60 (37;.5) Gy(RBE)PTV: planning target volume,RBE, relative biological effectiveness.

### Bladder volume (BV) measurements

The baseline BV was defined as obtained from TPCT images (BV_TPCT_), calculated by contouring the water dense area in the bladder. Just before the initiation of the treatment, the BV was measured three times with an ultrasound device (Bladder scan BVI9400, Verathon) on the patient couch of the SSPT system. The mean of these three measurements was defined as the BV at the time of treatment (BV_Treatment_). The vendor specifications for the nominal accuracy of the BV measurement is ±15%. Previous studies suggested strong correlations between the volumes measured by bladder scan and the CT contoured volume in prostate radiotherapy patients.^24,28^ Since the bladder scan findings are reported to be influenced by the presence of ascites^29^ and possibly in highly obese patients.^30^ Therefore, we evaluated ascites by TPCT and obesity by the body mass index (BMI). All BV measurements were performed by the same person.

### Prostate position measurements

Initially, registration between the treatment plan and daily set-up of the patient was performed with reference to the bone structure using two simultaneously obtained, orthogonal sets of diagnostic X-ray fluoroscopy images along the anteroposterior and lateral axes. Then, the distance from the planned to the actual position of the fiducial markers was measured in the Left-Right (LR), Cranial-Caudal (CC), and Anteroposterior (AP) directions using the two sets of X-ray fluoroscopy. The distance represents the prostate motion relative to the bone structure and includes some degree of uncertainty in bone matching, this distance was termed the prostate motion. The details of this method were reported in a previous study.^31^

### Analysis of the correlation between BV changes and prostate motion

The amount of BV changes was defined as the BV_Treatment_ minus BV_TPCT_. The linear correlation between the BV change and prostate motion was assessed by a linear mixed-effects model. The significance level was set at *p* < 0.05. The statistical analysis was performed using JMP Pro (v. 16, SAS institute, Cary, NC, USA).

If there is a non-linear correlation with a threshold between the BV change and prostate motion, the linear model may not show significance, and we also calculated the systematic error (Σ) and random error (σ) of the prostate position according to the reproducibility of BV and examined whether there was a threshold value for improving the reproducibility of the prostate motion. For a patient *i*, the mean value (m*_i_*) and standard deviation (σ*_i_*) of the prostate motion were calculated from all of the measurements. The Σ and σ were calculated as the standard deviation of m*_i_* and the root mean square of σ*_i_* among all patients in our cohort. As a provisional threshold of BV change from the BV_TPCT_, both absolute BV changes (less than 50 ml, 100 ml, 150 ml, and 200 ml) and relative BV changes (less than 50%, 100%, 150 and 200%) were evaluated.

## Results

### Patient characteristics and data sets

Details of patient characteristics and dose volume statistics are shown in Table 2. There were two obese patients (BMI 27.7 and 35.1) and none of the other patients exceeded BMI 25. None of the patients had significant ascites on the TPCT images. All treatment plans met dose constraints for both target and organs at risk within acceptable criteria. Ten patients were treated with SFUD and the remaining two were treated with IMPT. The follow-up period for patients from the start of radiotherapy was from 4.57 to 5.15 years (median 4.86 years).

**Table 2.** Patient characteristics and dose volume histogram parameters

Median age (years) (range)		63 (46–75)
**Median number of BV measurements (range**)		27 (8–30)
**Mean BMI (range**)		23.9 (17.7–35.1)
**Mean BV at TPCT (ml) (range) (1 SD**) **Median of mean BV at the time of treatment (ml) (range**) **Median of mean BV changes (ml) (range**)		78.9 (29.4–196.1) (58.8) 79.5 (24.9–142.9) 7.1 (−98.7- + 86.3)
**Tumor stage – No. (%**)	**T1c**	6 (50%)
	**T2a**	3 (25%)
	**T2b**	0 (0%)
	**T2c**	1 (8%)
	**T3a**	2 (17%)
**Hormone therapy – No. (%**)	–	6 (50%)
	**+**	6 (50%)
**NCCN risk classification**	**Low**	3
	**Favorable intermediate**	3
	**Unfavorable intermediate**	0
	**High**	6
**PTV**	**Mean D95Gy(RBE) (range**)	70.3 (70.0–71.0)
**CTV**	**Mean D99Gy(RBE)(range**)	73.1 (71.2–74.3)
	**Mean Dmax Gy(RBE)(range**)	76.2 (74.7–77.9)
**Rectum**	**Mean V60 Gy(RBE) (%) (range**)	17.6 (11.2–20.0)
	**Mean V37.5Gy(RBE) (%) (range**)	37.2 (28.7–43.2)
**Bladder**	**Mean V37.5Gy(RBE) (%)(range**)	23.1 (12.5–36.3)

BMI: body mass index,BV: bladder volume, CTV: clinical target volume, D95(99): the dose covering 95(99) % of the target, Dmax: max dose, NCCN: National Comprehensive Cancer Network, PTV: planning target volume,RBE, relative biological effectiveness; SD: standard deviation, TPCT: treatment planning computed tomography, V60(37.5)Gy(RBE): the volume receiving more than 60 (37..5) Gy(RBE)

### Clinical outcomes

Adverse events, acute and late GU and GI toxicities are shown in Table 3. The BV_TPCT_ and bladder V37.5Gy(RBE) in the two patients who experienced late Grade 1 GU toxicity (urinary urgency) was 196.1 ml and 12.6 %, and 190.7 ml and 12.7%, respectively. Two patients experienced late Grade 2 GI toxicity (rectal bleeding) during the follow-up. The BV_TPCT_, Bladder V37.5Gy(RBE), Rectum V37.5Gy(RBE) and V60Gy(RBE) of the two patients who experienced Grade 2 GI toxicity were 196.1 ml, 12.6%, 42.7%, and 20.0%; and 56.6 ml, 27.7 %, 36.0%, and 18.4%, respectively. Overall, there was no relationship between the GU/GI toxicity and BV_TPCT_ or BV change.

**Table 3.** Acute and late genitourinary and gastrointestinal toxicities

Acute (within 90 days)– No. (%)	Grade0	Grade 1	Grade 2
Genitourinary toxicities			
Urinary frequency	4 (33.3)	8 (66.7)	0
Urinary incontinence	12 (100)	0	0
Urinary retention	11 (91.7)	1 (8.3)	0
Urinary tract pain	9 (75.0)	3 (25.0)	0
Urinary urgency	9 (75.0)	3 (25.0)	0
Hematuria	12 (100)	0	0
Gastrointestinal toxicity			
Proctitis	8 (66.7)	4 (33.3)	0
Late (after 90 days)– No. (%)	Grade0	Grade 1	Grade 2
Genitourinary toxicities			
Hematuria	11 (83.3)	1 (8.3)	0
Urinary urgency	10 (83.3)	2 (16.7)	0
Gastrointestinal toxicity			
Rectal hemorrhage	4 (33.3)	6 (50.0)	2 (16.7)

One high-risk patient showed prostate-specific antigen (PSA) elevation during adjuvant hormone therapy (luteinizing-hormone releasing hormone agonist) after the SSPT and salvage hormone therapy (complete androgen blockade) was initiated before the PSA value elevated above the nadir +2 ng ml^−1^. One patient died of another disease (pneumonia) at 4.72 years after SSPT. All other patients survived without recurrence until the final observation period. No relationship was observed between tumor control and BV_TPCT_ or BV changes.

### Correlation between bladder volume and prostate motion

**Figure 2.**
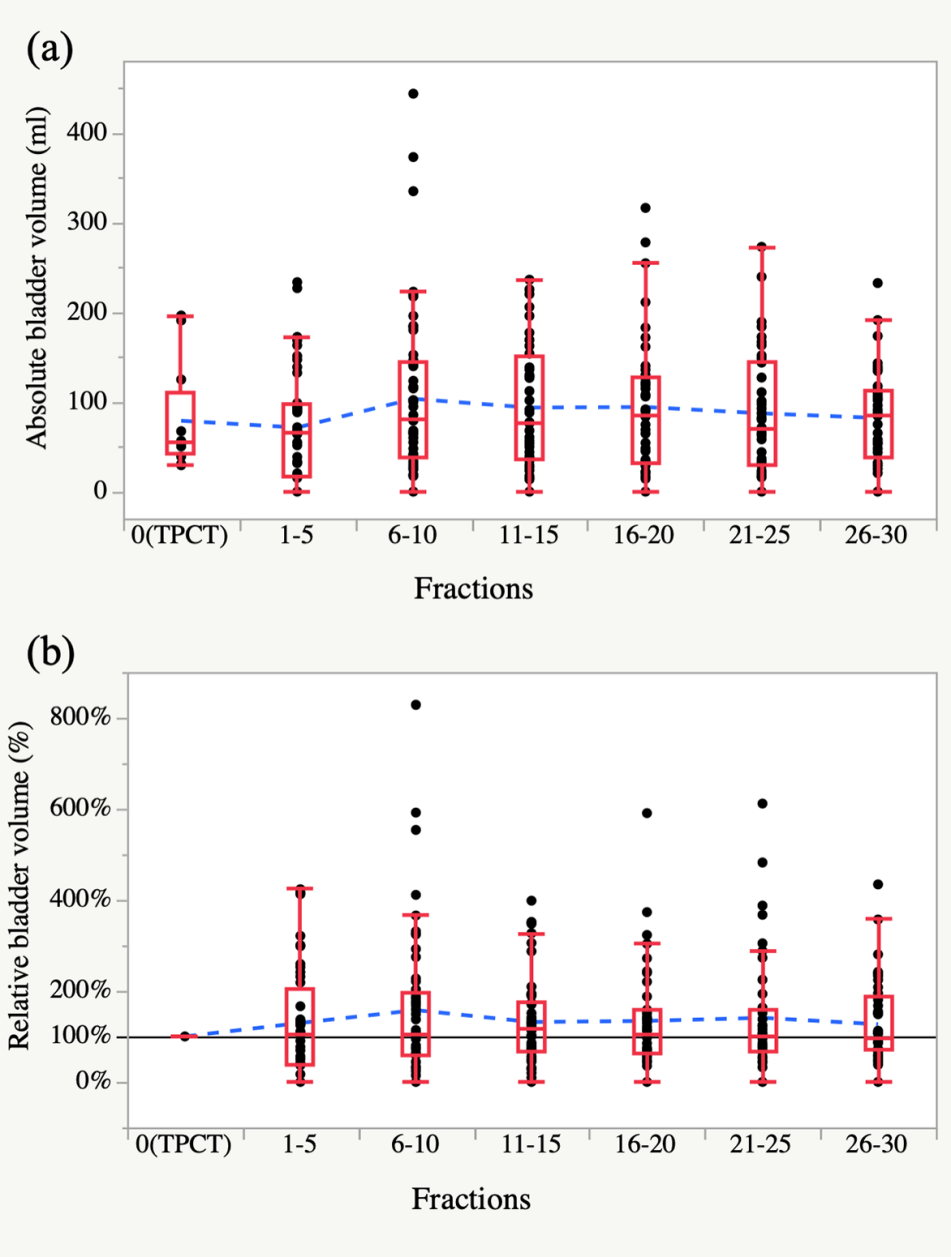
Absolute (**a**) and relative values of BV (**b**) from the treatment planning CT (TPCT) till the end of the treatment (30 fractions). In the box plots, the center line in the box indicates the median, and the upper and lower limits of the box indicate the third and first quartiles, respectively. The maximum and minimum of the whiskers indicate the “third quartile +1.5×IQR” and the “first quartile +1.5×IQR”. The horizontal dashed line is drawn to connect the mean BV values at the respective TPCT fractions. BV: bladder volume, IQR: interquartile range

The details of the BV_TPCT_, BV_Treatment_, and BV changes are shown in Table 2. The distribution of absolute BV_Treatment_ of all patients did not show any significant trend during the treatment course (Figure 2a). The mean relative BV change for the patients was from 48.3 to 252.6% (median 118.0 %). The distribution of relative BV in % also did not show any significant trend throughout the treatment (Figure 2b).

The mean absolute prostate motion from the TPCT position (range of SD in the 12 patients) was from −1.46 to 1.85 mm (0.21 to 0.60 mm), −6.10 to 3.65 mm (0.77 to 2.52 mm), and −1.90 to 5.23 mm (0.58 to 2.19 mm) in the LR, CC, and AP directions, respectively. The positive values indicate that the prostate moved to the left, cranial, and anterior directions in a patient.

**Figure 3.**
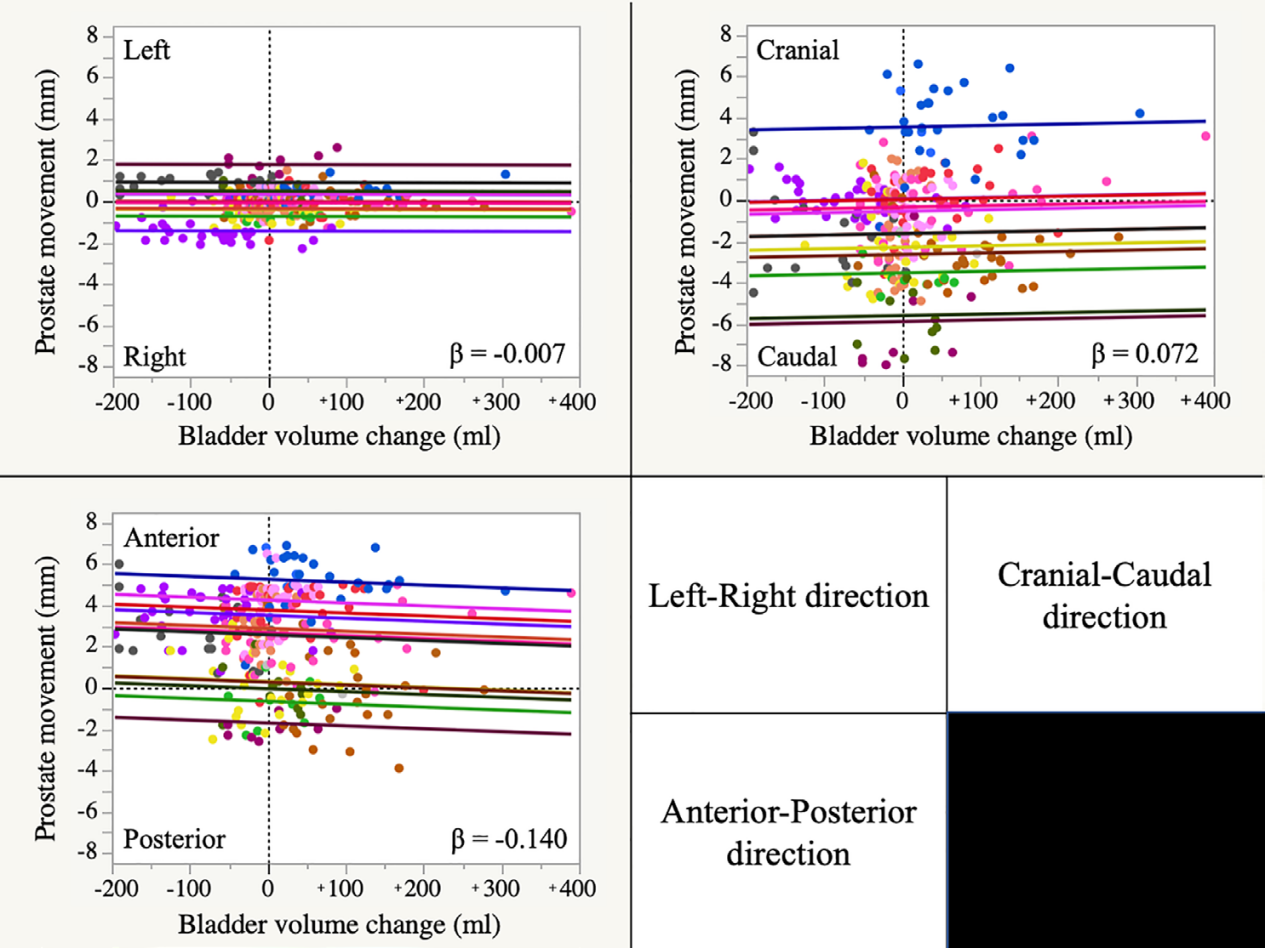
Regression plot graphs of the linear mixed effect model The horizontal axis shows the change in bladder volume from the baseline and the vertical axis shows the movement of the prostate. The color of the data points and fitting lines are different for each patient. The positive values of the vertical axis indicate that the prostate moved to the cranial, left and anterior directions in the patients, respectively. β: estimated coefficient of regression (mm/100 ml)

The linear mixed effects model showed no significant relationship between the BV change and prostate motion in any direction (Figure 3). The detailed results are shown in Table 4. The largest estimated coefficient of regression (β) was found in the AP direction as −0.140 mm/100 ml (95% confidence interval (CI) was from −0.414 to 0.134 mm/100 ml), and still it showed almost no clinical relationship.

**Table 4.** Estimated coefficient of regression and 95% confidence interval derived from the linear mixed effect model

Left-Right			Cranial-Caudal			Anteroposterior		
β	p	95% CI	β	p	95% CI	β	p	95% CI
−0.007	0.88	−0.099 to 0.086	0.072	0.65	−0.243 to 0.386	−0.140	0.31	−0.414 to 0.134

CI, confidence interval (mm/100ml); β, estimated coefficient of regression (mm/100ml).

The Σ and σ according to the BV change reproducibility are shown in Table 5. Both Σ and δ of the prostate motion were very little changed regardless of the amount of BV change. The results were the same whether the threshold value was the absolute amount or the relative amount of BV change. These data suggest that there was no apparent non-linear correlation within the range of the BV change and prostate motion.

**Table 5.** Systematic (Σ) and random errors (σ) (mm) according to the BV change reproducibility

		Absolute BV changes					Relative BV changes				
		<50 ml	<100 ml	<150 ml	<200 ml	Overall	-50%– +50%	-100%– +100%	-100%– +150%	-100%– +200%	Overall
No. of sessions		162	217	245	262	268	126	211	228	239	268
LR	Σ	0.800	0.862	0.848	0.839	0.841	0.810	0.806	0.817	0.840	0.841
	σ	0.471	0.487	0.469	0.464	0.464	0.430	0.464	0.478	0.479	0.464
CC	Σ	2.526	2.637	2.678	2.668	2.674	2.395	2.628	2.666	2.643	2.674
	σ	1.761	1.626	1.604	1.692	1.694	1.799	1.774	1.733	1.694	1.694
AP	Σ	2.307	2.25	2.264	2.289	2.275	2.174	2.251	2.255	2.243	2.275
	σ	1.447	1.389	1.336	1.362	1.367	1.517	1.437	1.413	1.397	1.367

AP, anterior-posterior; BV, bladder volume; CC, cranial-caudal; LR, left-right.

The CBCT was acquired in six sessions for five patients when a large prostate motion was observed. The relative value of the average rectal CSA and BV based on the area of the TPCT was from 76.4 to 111.8% (median 104.7%) and 21.4 to 371.6% (median 133.4%), respectively. No obvious CSA change was observed in the rectum. In four sessions for three patients with no change in volume observed in either rectum or bladder, it may be speculated that the cause of the prostate motion could be changes in muscle tone in the pelvic floor.

## Discussion

Recently, the effect of BV and BV changes on the prostate position is one of the active debates in the field of X-ray radiotherapy for prostate cancer.^15,17,18,24,32–37^ Theoretically, if BV or BV changes impact on the internal displacement of the prostate, it will obscure the potential benefits of cutting-edge external-beam radiotherapy, such as image-guided SSPT in relation to the conventional treatment.^9–13^

In the present study, neither Grade 3 or more early and late GU and GI toxicity nor PSA recurrence except for one clinical recurrence was observed in the follow-up period after the image-guided SSPT. We have also seen no impact of BV and BV changes on the early and late clinical outcomes. Regarding the relationship between BV_TPCT_ and BV changes, the amount of the absolute BV change was smaller in our present series compared to previous series.^24^ This finding, that small BV_TPCT_ resulted in small BV changes, is consistent with many previous studies which reported that the smaller BV_TPCT_ in ml is associated with less BV change in %.^18,38–40^

Although we have seen small BV in this study compared to the previous reports in X-ray therapy, the early and late GU and GI toxicity was minimal. Recently, Byun et al reported that BV had no impact on early and late GU and GI toxicities in the prostate hypofractionated stereotactic body radiotherapy (SBRT).^41^ Chetiyawardana et al also reported findings about the bladder filling for patients with localized prostate cancer after image-guided VMAT administered 60 Gy in 20 fractions with a median follow-up of 48 months (range 36–60 months). They found non-inferiority of an empty bladder filling protocol comparing to a full bladder filling protocol in respect to biological progression-free survival and GU and GI toxicities.^42^ Our clinical findings in image-guided SSPT were consistent with the reports of X-ray therapy from Byun et al and Chetiyawardana et al and, therefore, a bladder-emptying protocol could be preferable by virtue of reducing BV variation. Past studies which found correlations between BV and clinical outcome is for early GU toxicity and needs to be evaluated further for late toxicities but the inclusion of patients who received irradiation or surgery of the pelvis in their series may be related to the correlation between BV and GU toxicity.^23,36,40^

In X-ray therapy, Stam et al have published a study which investigated the relationship between BV changes and prostate position using BVI 3000 Bladder scan and fiducial markers in the prostate and found no correlation between BV changes in ml and the prostate motion.^24^ Stam et al have also shown that simple instructions are sufficient without any further intervention. The results in this study confirm the findings by Stam et al that there was no apparent relationship between BV changes and prostate position during fractionated radiotherapy. However, Pang et al have reported that by using instruction and education, CBCT, and daily transperinel ultrasound BV measurement, BV larger than 200 ml was weakly associated with prostate motion and that there was a reduction in prostate motion in both the CC (*p* = 0.008) and AP (*p* = 0.0001) directions when the daily bladder was filled between 82 and 113% (3rd Quartiles), independent of the BV_TPCT_.^36^ It is notable that the Pang et al study included 57.7% of patients who received whole pelvic irradiation before image-guided IMXT or volumetric modulated arc therapy (VMAT) whereas the Stam et al and the present report do not include patients who received pelvic irradiation. Our results cannot refute a relationship between the BV changes and prostate motion for the whole possible range of BV under instruction to the patient but the impact of BV changes on the prostate motion is suggested to be insignificant in the range of BV which we observed and involving just verbal instruction.

As other ways to improve the reproducibility of the prostate position, a rectal balloon would help to immobilize the prostate and reduce intrafractional prostate movements, however, this would not reduce interfractional variations.^43^ Since the number of sessions of our treatment was large, we did not use a rectal balloon in consideration of the invasiveness for the patients by balloon insertion. However, in the setting of ultrahypofractionated radiotherapy where the number of treatments is small, the dose of one treatment is large, and the treatment time is long, the insertion of a rectal balloon is a preferable method to suppress intrafactional movement of the prostate. In addition, the spacer gel that could not be used at the time of this study is nowadays available. This may also be useful for improving the reproducibility of the prostate position, however, previous studies showed no significant improvement in prostate immobilization by spacer gel implantation.^44,45^

The primary limitations of this study are the small number of patients and the retrospective nature. Another limitation is that we have no information about rectal volume, the deformation and rotation of the prostate, SV, and other adjacent OAR during fractionated SSPT. An evaluation using CBCT or CT imaging would be needed to accurately assess the motion of SV and whether the entire target is still covered sufficiently if the BV is changed. In the study we used a strict protocol for rectal volume control, however factors that change the position of the prostate other than the changes in rectal and bladder volume need to be investigated further.

## Conclusions

The present study suggests that limited simple verbal instructions about urination is sufficient to control the BV and BV change not to impact on the prostate motion and clinical outcome in image-guided SSPT. However, careful attention to BV change is still needed when the SV is to be treated as CTV. The small variations in BV recorded in this study provide support to reduce uncertainties in determining the length of passage of the proton beam from the viewpoint of dose distribution in SSPT. Further research will be needed to identify other factors that could affect the daily prostate position and ways to reduce uncertainties in the dose distribution.
